# Time Taken to Detect and Respond to Polio Outbreaks in Africa and the Potential Impact of Direct Molecular Detection and Nanopore Sequencing

**DOI:** 10.1093/infdis/jiab518

**Published:** 2021-10-08

**Authors:** Alexander G Shaw, Laura V Cooper, Nicksy Gumede, Ananda S Bandyopadhyay, Nicholas C Grassly, Isobel M Blake

**Affiliations:** Medical Research Council Centre for Global Infectious Disease Analysis, School of Public Health, Imperial College London, London, United Kingdom; Abdul Latif Jameel Institute for Disease and Emergency Analytics, School of Public Health, Imperial College London, London, United Kingdom; Medical Research Council Centre for Global Infectious Disease Analysis, School of Public Health, Imperial College London, London, United Kingdom; Abdul Latif Jameel Institute for Disease and Emergency Analytics, School of Public Health, Imperial College London, London, United Kingdom; World Health Organization African Region Office, Brazzaville, Congo; Bill and Melinda Gates Foundation, Seattle, Washington, USA; Medical Research Council Centre for Global Infectious Disease Analysis, School of Public Health, Imperial College London, London, United Kingdom; Abdul Latif Jameel Institute for Disease and Emergency Analytics, School of Public Health, Imperial College London, London, United Kingdom; Medical Research Council Centre for Global Infectious Disease Analysis, School of Public Health, Imperial College London, London, United Kingdom; Abdul Latif Jameel Institute for Disease and Emergency Analytics, School of Public Health, Imperial College London, London, United Kingdom

**Keywords:** poliovirus, direct detection, VDPV, outbreak, stool, AFP, nanopore, surveillance

## Abstract

**Background:**

Detection of poliovirus outbreaks relies on a complex laboratory algorithm of cell-culture, polymerase chain reaction (PCR), and sequencing to distinguish wild-type and vaccine-derived polioviruses (VDPV) from Sabin-like strains. We investigated the potential for direct molecular detection and nanopore sequencing (DDNS) to accelerate poliovirus detection.

**Methods:**

We analyzed laboratory data for time required to analyze and sequence serotype-2 VDPV (VDPV2) in stool collected from children with acute flaccid paralysis in Africa (May 2016–February 2020). Impact of delayed detection on VDPV2 outbreak size was assessed through negative binomial regression.

**Results:**

VDPV2 confirmation in 525 stools required a median of 49 days from paralysis onset (10th–90th percentile, 29–74), comprising collection and transport (median, 16 days), cell-culture (7 days), intratypic differentiation quantitative reverse transcription PCR (3 days), and sequencing, including shipping if required (15 days). New VDPV2 outbreaks were confirmed a median of 35 days (27–60) after paralysis onset, which we estimate could be reduced to 16 days by DDNS (9–37). Because longer delays in confirmation and response were positively associated with more cases (*P* < .001), we estimate that DDNS could reduce the number of VDPV2 cases before a response by 28% (95% credible interval, 12%–42%).

**Conclusions:**

DDNS could accelerate poliovirus outbreak response, reducing their size and the cost of eradication.

## Teaser

Delays in detecting poliovirus result in larger outbreaks. Direct molecular detection of poliovirus in stool by nanopore sequencing is fast, easy to implement, and could substantially decrease the time taken to respond to poliovirus outbreaks, accelerating eradication.

Poliomyelitis surveillance is based on the reporting of acute flaccid paralysis (AFP) followed by collection of stool and testing for poliovirus. Laboratory processing of stool for clinical or AFP surveillance and sewage samples for environmental surveillance is conducted within the Global Polio Laboratory Network (GPLN), a standardized network of 146 World Health Organization (WHO) accredited laboratories with regular quality control evaluations to maintain the highest standards of practice. This network is essential to process the approximately 100000 stool samples collected yearly from AFP surveillance.

For each AFP case, 2 stool samples are collected and sent to GPLN national polio laboratories (or a regional reference laboratory if a country lacks a national laboratory) for culture within susceptible cell lines, the current gold standard methodology for poliovirus detection [1]. Positive results require at least 6 days for the 2 WHO-recommended rounds of cell culture [2], with 14 days of culture required to confirm a negative result. Intratypic differentiation (ITD) via quantitative reverse transcription polymerase chain reaction (RT-qPCR) is performed on poliovirus isolates, with those having diverged from the Sabin strain, or being of serotype 2, or wild-type virus requiring Sanger sequencing at a global sequencing laboratory.

Whilst highly sensitive, this process is slow and complex, and can lead to delays in outbreak detection and response. This is especially important for serotype 2 vaccine-derived poliovirus (VDPV2), which is spreading rapidly across Africa following withdrawal of the serotype 2 oral poliovirus vaccine (OPV) from routine immunization in April 2016 [3, 4]. VDPVs can occur when the OPV circulates in populations with persistently low poliovirus immunity. Loss of attenuating mutations over time can yield strains that revert to pathogenicity [5]. To differentiate VDPV from Sabin OPV strains, allowing confirmation of a VDPV and consideration of a response, the approximately 900-bp VP1 capsid region must be sequenced. Many African countries do not have the capacity to perform the required sequencing, hence viral isolates often require shipping internationally once, if not twice, during testing. The nature of delays in sample testing experienced within the laboratory network and their impact on outbreak size and duration is unclear.

Direct detection methods have been developed (but not yet implemented by the GPLN) to increase the speed of detection and to remove the need for cell culture and replication of poliovirus, in line with containment targets [6, 7]. To combine both direct detection and the generation of a sequence, we recently employed direct molecular detection and nanopore sequencing (DDNS) of a nested VP1 PCR product direct from stool samples, observing a sensitivity of 88% to 99% across serotypes when compared to cell culture [8]. This method generates a full-length VP1 sequence and can be performed in only 3 days. Nanopore sequencing is highly portable and requires little laboratory support [9], so could be performed within the countries where stools are collected, reducing the need for stool and isolate shipping.

Here we assess the variation in the time to detect and sequence polioviruses across all countries reporting to the WHO African Regional Office, comprising the countries where the majority of VDPV2 outbreaks have occurred since 2016 [3]. We identify the drivers of these time intervals and how they differ by country. We explore the potential time savings from DDNS for the Global Polio Eradication Initiative. We quantify the potential impact of these time savings by assessing their association with the final size of outbreaks.

## METHODS

### Data

Children with AFP were reported through the routine polio surveillance network [10] and 2 stool samples were collected, ideally within 14 days of paralysis onset. Stool samples processed in laboratories reporting to the WHO African Regional Office were analyzed using data from the Polio Information System [11]. Samples from contacts and healthy children were excluded. AFP cases were restricted to those with paralysis onset between 1 May 2016 and 29 February 2020. These dates were chosen to analyze samples collected after OPV2 withdrawal when all serotype 2 positive samples were required to be sequenced. To characterize the stool testing process under normal operating conditions, we excluded cases that occurred during the coronavirus disease 2019 (COVID-19) pandemic.

### Statistical Analysis

**Figure 1.**
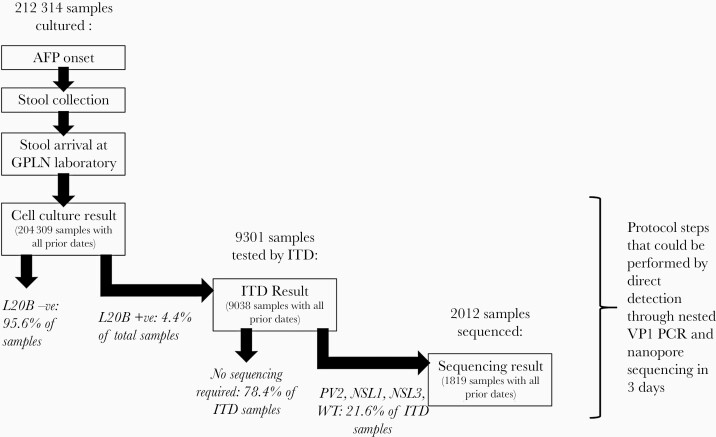
Data availability for keys steps in the current method of poliovirus detection from stool samples. Cell culture result, ITD result, and sequencing results are the 3 potential end points of testing, with only a subset of samples requiring ITD (those demonstrating a cytotoxic effect of L20B cells, indicating the presence of poliovirus [L20B +ve]), and a subset of those requiring sequencing (where ITD indicates a wild-type virus, serotype 2, or a non-Sabin serotype 1 or 3). Within each end point is shown the number of samples that had dates in chronological order for all the steps required to reach that end point. Abbreviations: AFP, acute flaccid paralysis; GPLN, Global Polio Laboratory Network; ITD, intratypic differentiation; PCR, polymerase chain reaction.

Stool samples are processed either until the completion of cell culture, ITD, or the sequencing of poliovirus isolates (Figure 1). For analysis of sample progression to each of these end points, only samples with recorded dates for all intermediate steps were included (Supplementary Table 1 for completeness of data). Time intervals were calculated between steps shown in Figure 1. Duplicate samples from a single case were retained for these calculations. Median values for intervals are presented with 10th and 90th percentiles. Comparisons between intervals used the Wilcoxon signed-rank test.

We assessed if testing intervals were associated with laboratory workload, allowing for differences in speed of processing by individual laboratories, through fitting a mixed-effects regression model in R version 4.0.0 [12], using the lme4 package [13]:


$$\begin{array}{l} x_{i,j}=\beta_{0}+\beta_{1}n_{i,j}+\mu_{j}+\varepsilon_{ij}\;\;\;\;\;\;\;\;\;I_{ij}\;\;\;\;\;\;\;\;\;\;\;\;\;\;\;\;\;\;\;\;\;\;\;\;\;\;\; \end{array}$$ (1)

$$\begin{array}{l} \mu_{j}∼Norm\left(0,\sigma_{j}^{2}\right) \end{array}$$

$$\begin{array}{l} \varepsilon_{ij}∼Norm\left(0,\sigma_{ij}^{2}\right) \end{array}$$

Where $x_{i,j}$ corresponds to the median interval for 1 of the 3 testing steps (cell culture, ITD, sequencing) for samples in month i, processed in lab j, and $n_{i,j}$ corresponds to the number of samples undergoing that test in laboratory j, in month i. The parameter $\mu_{j}$ corresponds to a normally distributed random effect of laboratory *j* to account for underlying differences in processing capacity of each laboratory. Importance of sample burden was assessed through a likelihood ratio test.

### Potential Impact of DDNS

We predicted testing intervals for optimal performance of the current methodology, assuming 6 days for 2 sequential rounds of cell culture from sample receipt for positive samples, plus 1 day for ITD. We compared these intervals to the estimated time required for DDNS, assuming sample processing immediately upon receipt in a GPLN laboratory and generating a detection and sequencing result in 3 days as previously demonstrated [8]. We predicted the impact of employing DDNS within the country of stool origin for countries that currently ship samples internationally for testing. In doing so, we assumed sample transport from origin to the laboratory required the same amount of time as countries currently performing cell culture in their national laboratories (median 2 days).

We estimated the potential benefits of DDNS on VDPV2 outbreak notification through comparing the notification date of each VDPV2 outbreak per country to a predicted date via DDNS (assuming 90% sensitivity per stool to detect VDPV2 compared with cell culture, as reported [8]). We generated a distribution of potential DDNS notification dates per VDPV2 outbreak (genetic lineage) per country by simulating whether each VDPV2 case would be detected by DDNS, taking a random draw from a Bernoulli distribution with a 90% probability of detection. We subsequently identified the earliest positive detection as the date of outbreak notification. One thousand simulations were performed per country outbreak. Where DDNS failed to detect outbreaks, notification was assumed to occur on the observed date generated by current methods.

We estimated the impact of timely outbreak response on VDPV2 burden by modelling the number of cases of VDPV2 paralysis per outbreak lineage per country occurring in the time from the first onset to the first vaccination response as a function of the number of days from first paralysis onset to first response. We allowed an additional 30 days for vaccination to take effect to reflect the time typically required to produce an immune response to the vaccine [14, 15]. We assume that the number of cases $Z_{ij}$ in country i caused by outbreak lineage j follows a negative binomial distribution with dispersion parameter k and expectation $q_{ij}$:


$$\begin{array}{l} Z_{ij}∼Negative\;\;\;Binomial\left(q_{ij},\;\;\;k\right) \end{array}$$

$$\begin{array}{l} log\left(q_{ij}\right)=\beta_{0}+\beta_{1}I_{ij}\;\;\;\;\;\;\;\;\;\;\;\;\;\;\;\;\;\;\;\;\;\;\; \end{array}$$ (2)

Where $I_{ij}$ is the number of days from the onset of the first case to the first vaccination response. We chose a negative binomial distribution because the total number of cases is a Poisson process subject to overdispersion from unobserved sources of variation in outbreak size. We exclude outbreaks that were first detected in environmental surveillance because we are considering the benefit of DDNS on stool. To test whether time to detection had a larger effect on case burden compared to the time to initiate the response, we fitted an additional model


$$\begin{array}{l} log\left(q_{ij}\right)=\beta_{0}+\beta_{1}K_{ij}+\beta_{1}L_{ij}\;\;\;\;\;\;\;\;\;\;\;\;\;\;\; \end{array}$$ (3)

Where $K_{ij}$ is the number of days from the paralysis onset of the first case to the time of notification and $L_{ij}$ is the number of days from notification to first vaccination response. The models were fitted to the data using the integrated nested Laplace approximation (INLA) approach, implemented in the R-INLA package [16]. The most parsimonious yet best fitting model was identified using the Watanabe-Akaike information criterion.

## RESULTS

### Reporting of Acute Flaccid Paralysis Cases and Overview of Stool Sample Process

We extracted the dates describing events in the processing of 212314 stool samples from 107463 individual AFP cases. These dates began with AFP onset and stool sample collection through to 3 potential end points, completion of cell culture, completion of ITD, or generation of a sequencing result according to whether poliovirus was detected and whether isolates required sequencing (Figure 1). Supplementary Table 2 shows the country of stool origin and where testing was performed.

### Time Intervals for Sample Processing and Drivers of Variation

**Figure 2.**
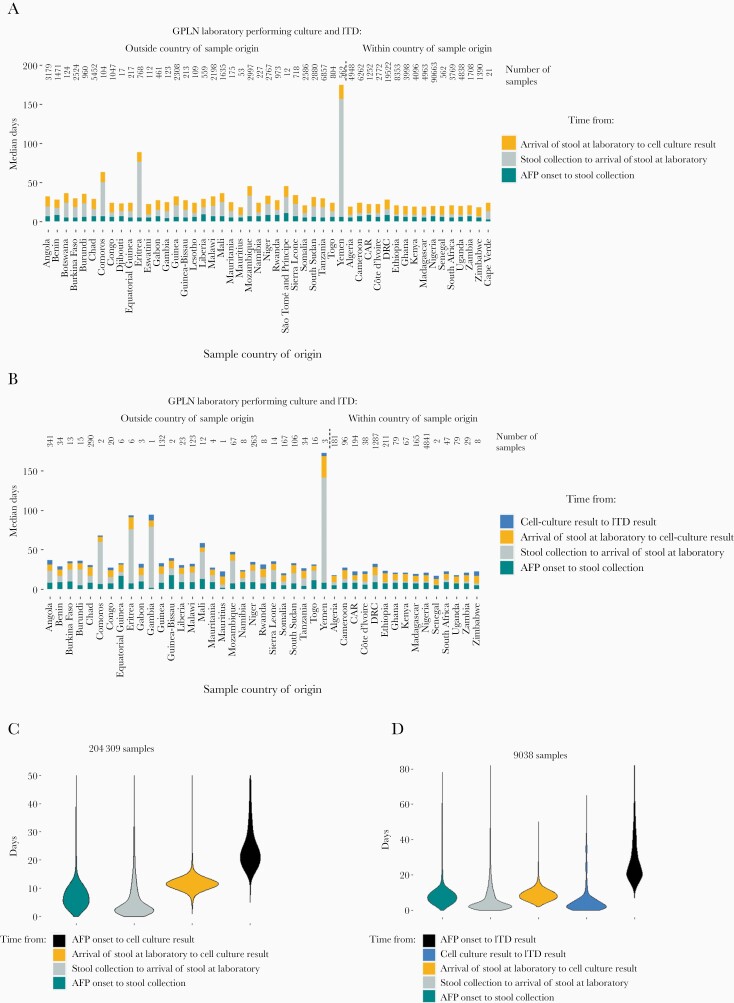
Time between steps in sample processing from AFP onset to cell culture or ITD results. The median time from AFP onset to (*A*) a cell culture result or (*B*) an ITD result is shown by country. The distribution of times taken for each step until (*C*) a cell culture result or (*D*) an ITD result is shown for all samples. *A* and *B*, Number above each bar indicates the number of samples originating from the country that were included in the analysis. Countries are grouped according to whether samples undergo cell culture and ITD within the country of origin or are shipped internationally for testing. Abbreviations: AFP, acute flaccid paralysis; GPLN, Global Polio Laboratory Network; ITD, intratypic differentiation.

Dates were recorded for all intermediate steps between AFP onset and a cell culture result for 204309 stool samples (Figure 2A and C). The WHO recommends that stool samples are attributed a cell culture result within 28 days of receipt by the laboratory [17], which occurred for 99.6% of samples. Processing in GPLN laboratories outside of the country of stool origin was performed for 45192 samples (22.1%) and resulted in significantly longer intervals between stool collection and arrival at the laboratory (median, 12 days vs 2 days, *P* < .001).

ITD was required for 9301 samples, and 9038 (97.2%) had dates for the 5 relevant processing steps (Figure 2B and D). The median time between ITD result and sequencing result was 14 days (10th–90th percentile, 7–29 days). For 63% of samples this included additional overseas shipping to arrive at 1 of 4 sequencing laboratories, involving a median of 3 days (1–6 days) after an ITD result prior to shipping and 6 days (4–13 days) before arriving at the sequencing laboratory. Processing intervals for all sequenced samples (n = 1819) are shown in Supplementary Figure 1.

We found evidence of a correlation between the number of samples requiring ITD or sequencing per month and the median time required for a result: there was an estimated increase of 3.09 days per 100 samples (95% confidence interval [CI], 2.03–4.16 days; *P* < .001) and 6.12 days per 100 samples (95% CI, −.53 to 12.7 days; *P* = .07), respectively (Supplementary Table 3).

### Observed Detection Intervals of VDPV2

**Figure 3.**
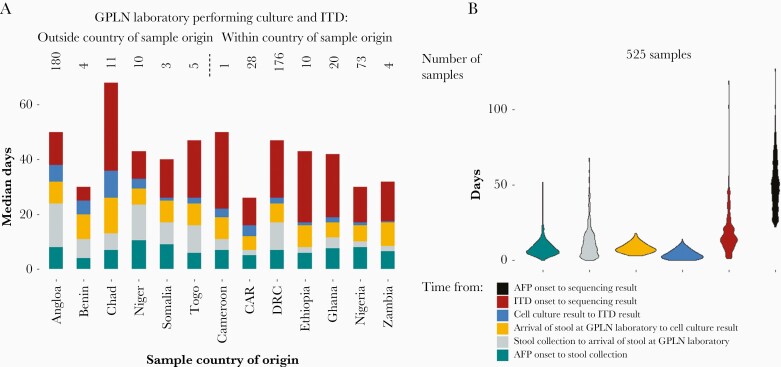
Time between steps in sample processing from AFP onset to a sequencing result for VDPV2-positive samples. *A*, Median time by country with the number above each bar indicating the number of samples originating from the country that were included in the analysis. Countries are grouped according to whether samples undergo cell culture and ITD within the country of origin or are shipped internationally for testing. *B*, Distribution of the time taken for each step and overall across all countries and samples. Abbreviations: AFP, acute flaccid paralysis; CAR, Central African Republic; DRC, Democratic Republic of the Congo; GPLN, Global Polio Laboratory Network; ITD, intratypic differentiation; VDPV2, serotype-2 vaccine-derived poliovirus.

Of the sequenced samples in the dataset, 525 contained VDPV2 and had dates for all stages of sample processing (92.4% of all sequenced VDPV2 samples). The median time between AFP onset and sequencing result was 49 days (10th–90th percentile range, 29–74 days); 16 days for collection and initial shipping, 7 days cell culture, 3 days ITD, and 15 days for shipping to a sequencing laboratory and subsequent sequencing (Figure 3). Of these samples, 94.7% were sequenced at a laboratory outside the country of stool origin, and 56% of samples had both ITD and sequencing performed in separate international laboratories.

### Estimated Benefits of Rapid DDNS Across All VDPV2 Cases

**Figure 4.**
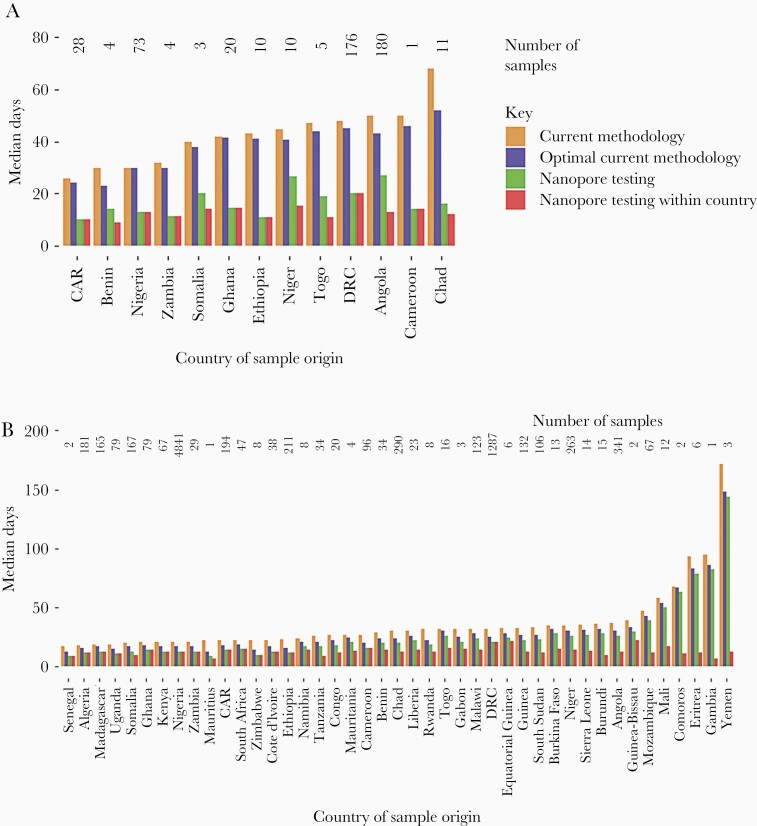
The potential impact of direct detection and poliovirus nanopore sequencing. Median intervals between AFP onset and (*A*) VDPV2-positive sequencing result and (*B*) ITD result by country. Observed intervals for the current methodology are compared with estimated intervals for the fastest possible implementation of cell culture, ITD, and sequencing, and for nanopore testing available only in GPLN laboratories or in all countries. Abbreviations: AFP, acute flaccid paralysis; CAR, Central African Republic; DRC, Democratic Republic of the Congo; GPLN, Global Polio Laboratory Network; ITD, intratypic differentiation; VDPV2, serotype-2 vaccine-derived poliovirus.

We predicted that DDNS would have reduced the interval between paralysis onset and VDPV2 confirmation from a median of 49 days (10th–90th percentile, 29–74 days) to 21 days (11–43 days). The largest gain would have been in Chad (52 days), and even in Benin and the Central African Republic this method would have reduced VDPV2 confirmation by 16 days (Figure 4A). We predict further gains if the method was performed in the country of stool origin (median estimated detection interval, 15 days [9–29 days]). Optimal operation of current methods would reduce confirmation by a median of 5 days.

### Estimated Benefits of Rapid DDNS Across All Poliovirus-Positive Stool Samples

DDNS is predicted to also reduce the time from AFP onset to ITD result for any poliovirus positive sample, with a median reduction from 25 days (10th–90th percentile range, 16–49 days) to 15 days (9–32 days), with the greatest reductions seen in Zimbabwe, Mauritius, and Ethiopia (Figure 4B). Testing within the country of stool origin would further reduce the median interval to 14 days (9–25 days), whilst optimal performance of current methods would reduce the interval to 19 days (13–36 days).

### Impact of Rapid DDNS on VDPV2 Outbreak Detection

**Figure 5.**
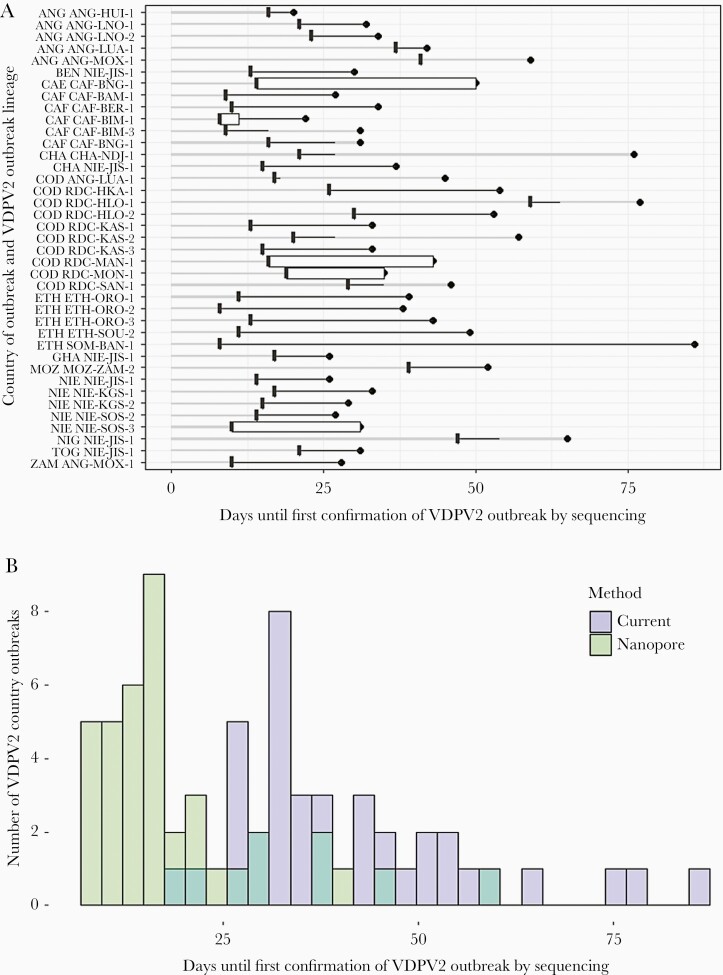
Predicted impact of direct detection and nanopore sequencing on the timeliness of detection of VDPV2 outbreaks. *A*, Observed number of days between the earliest onset of AFP and detection of each VDPV2 outbreak within a country according to current detection methods (filled circle and grey line) and the estimated number of days for this interval across outbreaks using the nanopore method with 9% sensitivity per sample (boxplots with 90% range, black line). Boxplot ranges are often narrow as an initial AFP case with 2 collected stool samples gives a 99% probability of outbreak detection. Y-axis indicates the country of the outbreak (World Health Organization acronym code) followed by the VDPV2 lineage. *B*, Distribution across all country outbreaks of the interval between AFP onset and confirmation by sequencing of each VDPV2 outbreak for the 2 detection methods. Abbreviations: AFP, acute flaccid paralysis; ANG, Angola; BEN, Benin; CAE, Cameroon; CAF, Central African Republic; CHA, Chad; COD, Democratic Republic of the Congo; ETH, Ethiopia; GHA, Ghana; MOZ, Mozambique; NIE, Nigeria; NIG, Niger; TOG, Togo; VDPV2, serotype-2 vaccine-derived poliovirus; ZAM, Zambia.

During the study period there were 38 unique VDPV2 outbreaks, with cases reported in 17 African countries, resulting in 56 country outbreaks. Ten did not report AFP cases and a further 9 were missing required laboratory dates, leaving 37 country outbreaks in 13 countries to study the potential impact of DDNS. The observed median interval between the first AFP onset and the first confirmation of a VDPV2 sequence from stool was 35 days (10th–90th percentile, 27–60 days). We estimate that DDNS would reduce this interval to 16 days (9–37 days) (Figure 5A and 5B, and Supplementary Table 4). We estimated that the greatest reductions would occur in Chad (39 days) and Cameroon (36 days). We estimate that a mean of 3 samples from each outbreak would yield a sequencing result by DDNS prior to outbreak confirmation by current methods. Assuming a per sample sensitivity of 90%, the probability of detecting the outbreak following testing of these 3 stool samples by DDNS would be 99.9%.

### Programmatic Benefits of Rapid DDNS

**Figure 6.**
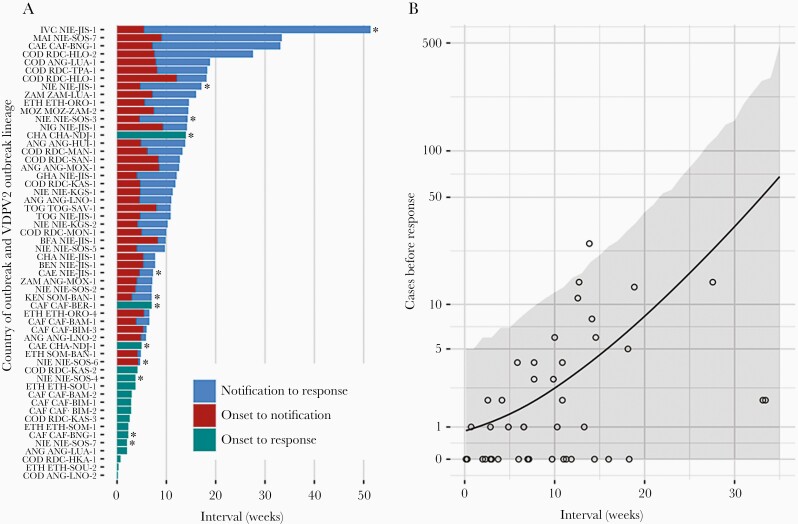
*A*, Interval in weeks between onset of AFP or environmental sample collection and notification to WHO headquarters (red) and between notification and vaccination response campaign (blue) for each outbreak in a given country. For outbreaks where vaccination took place before notification (because of a response to a nearby outbreak), overall interval between onset and response shown (green). Y-axis indicates the country of the outbreak (World Health Organization acronym code) followed by the VDPV2 lineage. Asterisks indicate outbreaks that were first detected by environmental surveillance. *B*, Number of VDPV2 cases between first isolate and 30 days after first response by interval between onset and response. Lines show mean expected cases, with 2.5th and 97.5th percentiles shown by ribbon. The y-axis is plotted on a pseudo-log scale. Abbreviations: AFP, acute flaccid paralysis; ANG, Angola; BEN, Benin; CAE, Cameroon; CAF, Central African Republic; CHA, Chad; COD, Democratic Republic of the Congo; ETH, Ethiopia; GHA, Ghana; MOZ, Mozambique; NIE, Nigeria; NIG, Niger; TOG, Togo; VDPV2, serotype-2 vaccine-derived poliovirus; ZAM, Zambia.

Rapid detection of VDPV2 outbreaks would allow for earlier responses, resulting in fewer cases. The number of days between first paralysis onset and first vaccination response in an outbreak was associated with the number of VDPV2 cases reported in the country during this interval (plus 30 days), with an average increase of 12% per additional week (95% credible interval [CrI], 5%–21%; Figure 6B). Assuming DDNS reduces VDPV2 detection time by 20 days (estimated above), we estimate a median 28% (95% CrI, 12%–42%) decrease in the mean number of cases occurring before the first vaccination response (Supplementary Tables 5–7). These results are based on 43 country outbreaks within WHO African Regional Office that were first detected in stool, with a vaccination response within 6 months following the last case (a further 13 country outbreaks occurred during the period of analysis but did not meet these conditions). The median interval from first paralysis onset to first vaccination response was 69 days (Figure 6A). In 11 outbreaks, the first vaccination response occurred before outbreak notification because of a response planned to another nearby confirmed outbreak. In the remaining 32 outbreaks, the median interval from onset to notification was 37 days and the median interval from notification to response was 42 days.

## DISCUSSION

Outbreaks of type-2 vaccine-derived poliovirus are threatening polio eradication. We show that the size of VDPV2 outbreaks is associated with the time taken for an outbreak response, which is in part determined by time taken to detect VDPV2. Outbreaks are primarily identified through a surveillance network for children with AFP, whose stools are tested for poliovirus through cell culture, ITD, and Sanger sequencing. This method is highly sensitive; however, we identify lengthy intervals between a case and confirmation, particularly for VDPVs where a sequence is required to confirm the virus and samples or viral isolates often need to be shipped internationally. DDNS can facilitate faster testing, providing both a result and a sequence in as little as 3 days, and requires minimal laboratory infrastructure [8].

The average time to confirm VDPV2 cases across the African Region was 49 days, with substantial variation between countries. Countries shipping samples to external regional reference laboratories face a significantly increased interval between sample collection and sample arrival in a GPLN laboratory. Of VDPV2-positive samples, 56% were shipped a second time to specialized global laboratories for sequencing, contributing to significant increases in time until sequence availability. DDNS can provide a solution to the demands of sample shipping for sequencing.

We find evidence that laboratory processing times for ITD and sequencing have risen as sample numbers requiring these assays have increased, albeit with variation between laboratories. There has been almost a 10-fold increase in the number of samples requiring sequencing between 2017 and 2019, from only 110 to 971. This increase is not only due to the expansion of VDPV2 transmission over time but also the high frequency of type 2 Sabin-like virus in stool following outbreak response campaigns with type-2 OPV. High-throughput DDNS, where tens of samples can be multiplexed using barcoded primers, can substantially reduce the per sample workload. The nested PCR protocol ensures a relatively narrow range of amplicon concentrations for samples testing positive, permitting rapid equal-volume pooling without wide deviations in per sample read depth. Live data analysis using RAMPART and the realtime-polio module further ensure that sufficient sequencing depths are achieved [8]. This pooling strategy reduces error associated with picking and equilibrating individual samples.

Relatively low capital costs and a simple protocol mean that DDNS can be employed at regional reference laboratories, and potentially even at national reference laboratories, further reducing the requirement for sample and isolate shipping. Even samples requiring only ITD will benefit from the combined effects of reduced shipping, high-throughput, and more rapid testing. Whilst the majority of stool samples being tested are poliovirus negative, rapid detection remains of value, with negative results confirmed within 3 days rather than 2 weeks (the minimum cell culture time required to confirm a negative result by WHO guidelines [17]).

We estimate that DDNS could have halved the time required for the sequence-based confirmation of VDPV2 outbreaks during the study period, despite assuming only 90% detection sensitivity per sample. For the 334 VDPV2-positive cases in our dataset, 67% yielded 2 stools positive by culture, mitigating the reduced sensitivity compared to culture because of the 2 opportunities to detect poliovirus. The rapid turnaround of DDNS allows for multiple samples to be processed prior to a sequencing result being available via cell culture.

We have calculated the proportion of the delay between a VDPV2 outbreak and a response that can be attributed to the detection of the outbreak. Once an outbreak is confirmed there is variation in the time taken to initiate the response campaign. DDNS of VDPV2 would have a sizeable impact on reducing the overall interval between the start of the outbreak and response, resulting in smaller outbreaks that are easier to contain.

There are some limitations to our analysis. We have only considered data from the WHO African Region. This Region does, however, include both the largest number of VDPV2 outbreaks and the greatest number of countries affected with on-going poliovirus transmission. We have also not considered the testing of stool from contacts, healthy children, and environmental samples. Stool samples from contacts and healthy children comprised 18% of the total stools collected in WHO African Region during the study period, but the processing could be performed by DDNS. The relative portability of the protocol and speed of detection could even allow novel methods of surveillance, facilitating localized testing around cases. DDNS can also be used on environmental samples, with RNA extraction being performed on viral concentrate, and work is ongoing to optimize the sensitivity of the method. When estimating the benefits of DDNS to VDPV2 detection we assumed 100% sensitivity by cell culture; this is unlikely to be the case [1]. Studies performing both cell culture and direct detection by qPCR in various scenarios have measured sensitivities below 100% for cell culture detection of poliovirus in stool [18–20]. We considered only the cases between outbreak onset and a response when estimating the impact of DDNS on outbreaks. Earlier responses could, however, reduce the probability of the virus escaping the response zone and thus lead to a greater reduction in cases.

DDNS has currently only been tested on stool samples in a single GPLN laboratory in Pakistan [8]. The time required to perform the protocol was estimated at a conservative 3 days, likely achievable by most laboratories. The sensitivity of the method was estimated at 90%, although automated RNA extraction platforms and increased sampling of stool volume through larger extractions and may increase this. Other key concerns prior to any use by the GPLN include the design of quality controls, accredited training of staff, and the development of infrastructure to disseminate the regionally generated sequence data through the GPLN. Additionally, identification of novel OPV2 will require the sequencing of a larger amplicon given the absence of mutations in the VP1 region that discriminate it from the Sabin strain [21].

We have explored the potential benefits of a single method of rapid, direct detection. Prior comparisons between cell culture and direct detection methods have largely been based on sensitivity. In this analysis we have found that the speed of the method also has a considerable impact. Other direct detection methods are being developed and methodological comparisons would benefit from a target product profile considering a range of factors including affordability, speed, ease of implementation, generation of a VP1 sequence, and accessibility of the technology to the eradication program.

Rapid detection of poliovirus will allow quicker responses to outbreaks of wild poliovirus and VDPVs. We estimated the performance of 1 of these methods and the potential impact that rapid detection could have if incorporated into global poliovirus surveillance. Whilst the adoption of this technology would require substantial support, the impact could be far-reaching for the Global Polio Eradication Initiative’s ability to interrupt ongoing chains of poliovirus transmission.

## Supplementary Data

Supplementary materials are available at *The Journal of Infectious Diseases* online. Supplementary materials consist of data provided by the author that are published to benefit the reader. The posted materials are not copyedited. The contents of all supplementary data are the sole responsibility of the authors. Questions or messages regarding errors should be addressed to the author.

jiab518_suppl_Supplementary_Table_S1

jiab518_suppl_Supplementary_Table_S2

jiab518_suppl_Supplementary_Table_S3

jiab518_suppl_Supplementary_Table_S4

jiab518_suppl_Supplementary_Table_S5

jiab518_suppl_Supplementary_Table_S6

jiab518_suppl_Supplementary_Table_S7

jiab518_suppl_Supplementary_Figure_S1

## Notes

***Acknowledgments.*** We thank Ashley L. Burman for data retrieval from POLIS and everyone involved in poliovirus surveillance and the Global Polio Laboratory Network, including the Ministries of Health from each country contributing data to this study.

***Financial support.*** This work was supported the Bill and Melinda Gates Foundation ( grant numbers OPP1171890 and OPP1207299); the Medical Research Council (MRC) Centre for Global Infectious Disease Analysis (grant number MR/R015600/1 to A. S., L. C., N. C. G., and I. B.) funded by the UK MRC and the UK Foreign, Commonwealth, and Development Office (FCDO), under the MRC/FCDO Concordat agreement and the EDCTP2 Program supported by the European Union; and Community Jameel. N. C. G. is supported by the World Health Organization Polio Research Committee.

***Potential conflicts of interest.*** All authors: No reported conflicts of interest. All authors have submitted the ICMJE Form for Disclosure of Potential Conflicts of Interest. Conflicts that the editors consider relevant to the content of the manuscript have been disclosed.

## References

[1] Wood DJ , Hull B. L20B cells simplify culture of polioviruses from clinical samples. J Med Virol 1999; 58:188–92. 10335869

[2] Martin EJ. Poliovirus methods and protocols. Springer protocols. Humana Press; 2016. doi:10.1007/978-1-4939-3292-4.

[3] Macklin GR , O’Reilly KM, Grassly NC, et al Evolving epidemiology of poliovirus serotype 2 following withdrawal of the serotype 2 oral poliovirus vaccine. Science 2020; 368:401–5. 32193361 10.1126/science.aba1238 PMC10805349

[4] Zambon M , Martin J. Polio eradication: next steps and future challenges. Euro Surveill 2018; 23:1800625. doi:10.2807/1560-7917.ES.2018.23.47.1800625. PMC6341938 30482261

[5] Stern A , Yeh MT, Zinger T, et al The evolutionary pathway to virulence of an RNA Virus. Cell 2017; 169:35–46.e19. 28340348 10.1016/j.cell.2017.03.013 PMC5787669

[6] World Health Organization. WHO global action plan to minimize poliovirus facility-associated risk after type-specific eradication of wild polioviruses and sequential cessation of oral polio vaccine use. http://polioeradication.org/wp-content/uploads/2016/12/GAPIII_2014.pdf. Accessed 12 February 2021.

[7] World Health Organization. Polio eradication strategy 2022–2026. https://polioeradication.org/wp-content/uploads/2021/06/polio-eradication-strategy-2022-2026-pre-publication-version-20210609.pdf. Accessed 1 August 2021.

[8] Shaw AG , Majumdar M, Troman C, et al Rapid and sensitive direct detection and identification of poliovirus from stool and environmental surveillance samples by use of nanopore sequencing. J Clin Microbiol 2020; 58:e00920-20. doi:10.1128/JCM.00920-20. 32611795 PMC7448630

[9] Quick J , Loman NJ, Duraffour S, et al Real-time, portable genome sequencing for Ebola surveillance. Nature 2016; 530:228–32. 26840485 10.1038/nature16996 PMC4817224

[10] World Health Organization. Polio: vaccine preventable diseases surveillance standards. https://www.who.int/publications/m/item/vaccine-preventable-diseases-surveillance-standards-polio. Accessed 1 November 2021.

[11] World Health Organization. Polio information system. https://extranet.who.int/polis/public/CaseCount.aspx. Accessed 20 August 2020.

[12] R Core Team. R: a language and environment for statistical computing. https://www.R-project.org. Accessed 1 November 2021.

[13] Bates D , Machler M, Bolker BM, Walker SC. Fitting linear mixed-effects models using lme4. J Stat Softw 2015; 67:1–48.

[14] Zaman K , Estívariz CF, Morales M, et al Immunogenicity of type 2 monovalent oral and inactivated poliovirus vaccines for type 2 poliovirus outbreak response: an open-label, randomised controlled trial. Lancet Infect Dis 2018; 18:657–65. 29571817 10.1016/S1473-3099(18)30113-0 PMC10495755

[15] Sáez-Llorens X , Bandyopadhyay AS, Gast C, et al Safety and immunogenicity of two novel type 2 oral poliovirus vaccine candidates compared with a monovalent type 2 oral poliovirus vaccine in children and infants: two clinical trials. Lancet 2021; 397:27–38. 33308427 10.1016/S0140-6736(20)32540-X PMC7811205

[16] Rue H , Martino S, Chopin N. Approximate Bayesian inference for latent Gaussian models by using integrated nested Laplace approximations. J R Stat Soc B 2009; 71:319–92.

[17] World Health Organization (WHO). Polio laboratory manual. 4th ed. Geneva, Switzerland: WHO, 2004.

[18] Taniuchi M , Begum S, Uddin MJ, et al Kinetics of poliovirus shedding following oral vaccination as measured by quantitative reverse transcription-PCR versus culture. J Clin Microbiol 2015; 53:206–11. 25378579 10.1128/JCM.02406-14 PMC4290924

[19] Moran-Gilad J , Mendelson E, Burns CC, et al Field study of fecal excretion as a decision support tool in response to silent reintroduction of wild-type poliovirus 1 into Israel. J Clin Virol 2015; 66:51–5. 25866337 10.1016/j.jcv.2015.03.005

[20] Giri S , Rajan AK, Kumar N, et al Comparison of culture, single and multiplex real-time PCR for detection of Sabin poliovirus shedding in recently vaccinated Indian children. J Med Virol 2017; 89:1485–8. 28213965 10.1002/jmv.24793 PMC6139431

[21] Yeh MT , Bujaki E, Dolan PT, et al Engineering the live-attenuated polio vaccine to prevent reversion to virulence. Cell Host Microbe 2020; 27:736–51.e8. 32330425 10.1016/j.chom.2020.04.003 PMC7566161

