# Supplementary material for: Time Taken to Detect and Respond to Polio Outbreaks in Africa and the Potential Impact of Direct Molecular Detection and Nanopore Sequencing
Source: J Infect Dis. 2021 Oct 8;226(3):453–62. doi: 10.1093/infdis/jiab518 (PMC9417130; doi:10.1093/infdis/jiab518)
Supplement: jiab518_suppl_Supplementary_Figure_S1 [file jiab518_suppl_supplementary_figure_s1.docx]

***Supplementary Figure 1 -* *Time between steps in sample processing from AFP onset to sequencing result for all sequenced samples.***


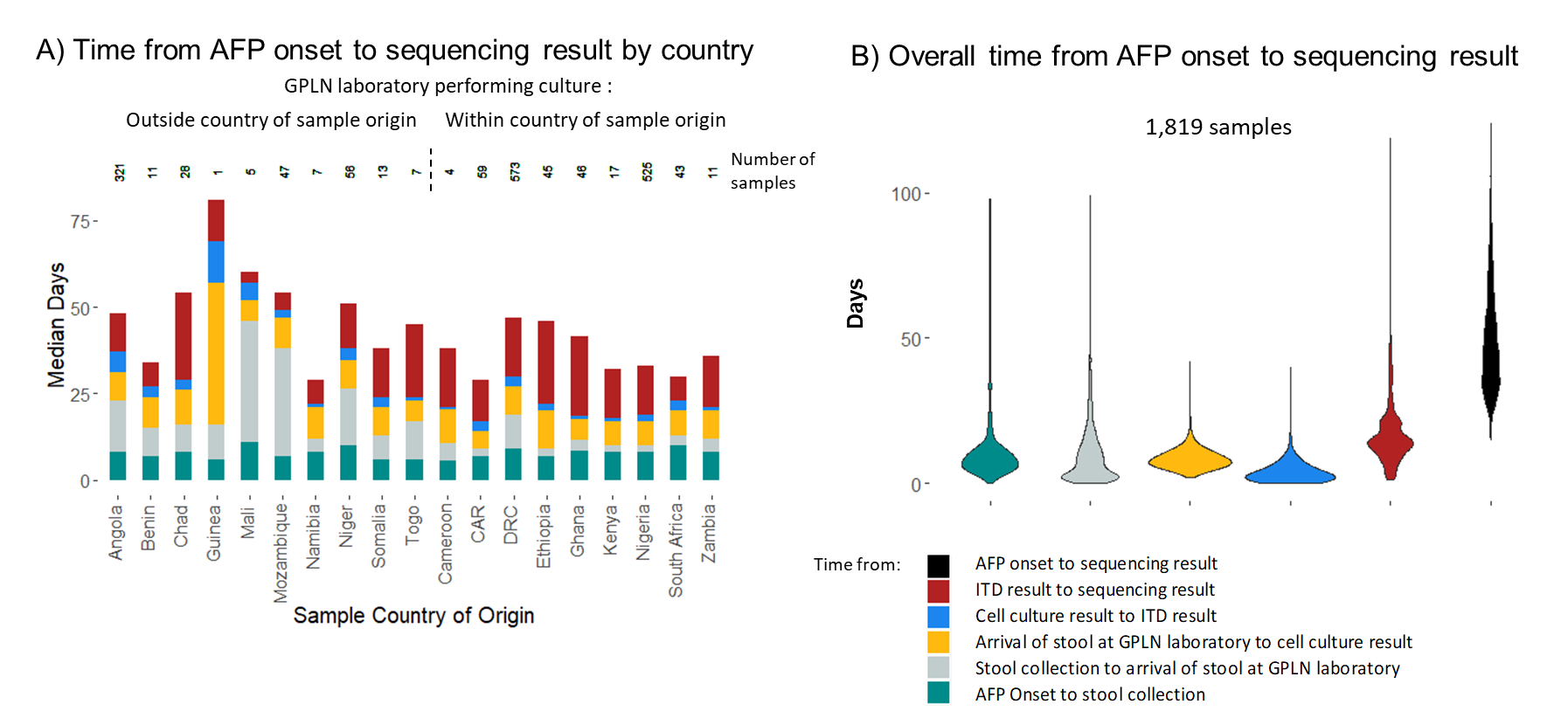


*Time between steps in sample processing from AFP onset to a sequencing result. In A) the median time by country is shown, with the number above each bar indicating the number of samples originating from the country that were included in the analysis and with countries grouped according to whether cell-culture is performed within that country or shipped internationally for testing. In B) the distribution of the time taken for each step and overall is shown as a violin plot, aggregating across all countries.*
